# Liver epithelial focal adhesion kinase modulates fibrogenesis and hedgehog signaling

**DOI:** 10.1172/jci.insight.141217

**Published:** 2020-10-15

**Authors:** Yun Weng, Tyler J. Lieberthal, Vivian X. Zhou, Maya Lopez-Ichikawa, Manuel Armas-Phan, Tristan K. Bond, Miya C. Yoshida, Won-Tak Choi, Tammy T. Chang

**Affiliations:** 1 Department of Surgery,; 2 Department of Pathology, and; 3 Liver Center, University of California, San Francisco, California, USA.

**Keywords:** Cell Biology, Hepatology, Fibrosis, Integrins, Signal transduction

## Abstract

Focal adhesion kinase (FAK) is an important mediator of extracellular matrix–integrin mechano-signal transduction that regulates cell motility, survival, and proliferation. As such, FAK is being investigated as a potential therapeutic target for malignant and fibrotic diseases, and numerous clinical trials of FAK inhibitors are underway. The function of FAK in nonmalignant, nonmotile epithelial cells is not well understood. We previously showed that hepatocytes demonstrated activated FAK near stiff collagen tracts in fibrotic livers. In this study, we examined the role of liver epithelial FAK by inducing fibrotic liver disease in mice with liver epithelial FAK deficiency. We found that mice that lacked FAK in liver epithelial cells developed more severe liver injury and worse fibrosis as compared with controls. Increased fibrosis in liver epithelial FAK-deficient mice was linked to the activation of several profibrotic pathways, including the hedgehog/smoothened pathway. FAK-deficient hepatocytes produced increased Indian hedgehog in a manner dependent on matrix stiffness. Furthermore, expression of the hedgehog receptor, smoothened, was increased in macrophages and biliary cells of hepatocyte-specific FAK-deficient fibrotic livers. These results indicate that liver epithelial FAK has important regulatory roles in the response to liver injury and progression of fibrosis.

## Abstract 2

Liver epithelial-specific FAK-deficient mice develop worse liver injury and fibrosis compared to controls, indicating that FAK has a regulatory role.

## Graphical abstract

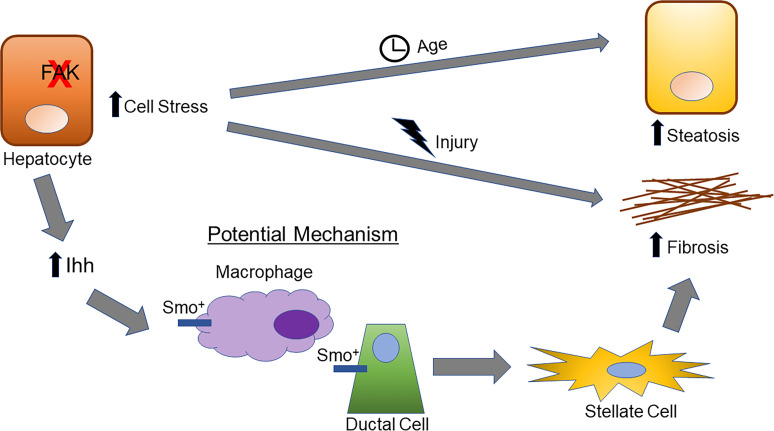

## Introduction

Mechano-signal transduction is the process through which physical stimuli are converted to biochemical signals within cells that orchestrate cell morphology and behavior. Focal adhesion kinase (FAK) is a key molecular mechano-sensor that connects the extracellular matrix with a cell’s cytoskeleton (1). Through its dual function as a tyrosine kinase and scaffolding protein, FAK mediates signals triggered by integrin binding to extracellular matrix ligands and links integrin activation, via adaptor proteins, to actin cytoskeletal responses (2). The downstream effects of FAK-mediated signals are multiple and include stimulation of cell motility, proliferation, and survival (3, 4). FAK also broadly interacts with and cross-regulates other mechano-sensors and mediators of integrin signals, including Src (5, 6), Rho family GTPases (7, 8), integrin-linked kinase (ILK) (9, 10), and Yes-associated protein (YAP) (11, 12).

FAK is overexpressed in many types of cancers. Elevated FAK expression or activation correlates with increased invasiveness, metastases, and poorer prognosis (13). Stiffened tumor stroma activates FAK and downstream cell contractility, which cooperate with growth factor–mediated signals to potentiate proliferation and invasive behavior of malignant cells (7, 8). Hepatocellular carcinoma is one cancer type that frequently demonstrates FAK overexpression and/or hyperphosphorylation (14). Culture of hepatocellular carcinoma cell lines on stiff matrices induces FAK activation and promotes proliferation and chemotherapy resistance (15). Moreover, deletion of FAK in hepatocytes repressed the development of a mouse model of hepatocellular carcinoma (16). Accordingly, FAK is thought to be a promising anticancer therapeutic target (17). Numerous FAK-specific small-molecule inhibitors have been developed (18), and several ongoing clinical trials are testing the safety and efficacy of FAK inhibition in the treatment of aggressive solid tumors in humans (19, 20).

In addition to proliferative and prosurvival effects, tumor-intrinsic FAK activation has been shown to amplify fibrotic stiffening of the peritumor stroma, resulting in bidirectional tumor stroma positive feedback of mechanics-induced signaling (21, 22). A stiffened microenvironment (23–25) and FAK activation (26, 27) are required for the differentiation of myofibroblasts, which are key fibrogenic effector cells that deposit pathologic collagen matrix. In chronic fibrotic disorders, FAK activity is elevated in intralesional skin fibroblasts from patients with systemic sclerosis (28) and in lung myofibroblasts from patients with idiopathic pulmonary fibrosis (29). FAK signaling is required for fibroblast and hepatic stellate cell durotaxis, in which motile cells migrate directionally toward more rigid matrix (12, 30, 31). FAK activation is required to induce lung myofibroblast profibrotic phenotype, and administration of FAK inhibitors in vivo attenuates the development of an experimental model of lung fibrosis (32). Likewise, FAK activation is required for hepatic stellate cell expression of α–smooth muscle actin and profibrotic collagens. Treatment with FAK inhibitors reduces the severity of carbon tetrachloride–induced liver fibrosis in mice (33). These preclinical studies have motivated considerable interest in developing FAK inhibitors as a novel therapeutic for fibrotic diseases (34). However, while activation of FAK signaling in myofibroblasts may be profibrotic, less is known regarding the function of FAK in parenchymal epithelial cells. It is possible that FAK serves important homeostatic functions in epithelial cells related to cell adhesion and mechano-sensing. Global inhibition of FAK with systemic inhibitor administration may demonstrate a predominant effect on suppressing myofibroblast activation but mask concurrent detrimental effects on epithelial homeostasis. In this study, in order to delineate potentially differential roles of FAK signaling in different cell types during fibrogenesis, we investigated the effect of liver epithelium–specific deletion of FAK on liver fibrosis.

There is evidence to suggest that tonic integrin- and FAK-mediated signals are important for maintaining liver homeostasis. Prolonged knockdown of β_1_ integrin in hepatocytes results in distorted bile canaliculi morphology and liver injury (35, 36). Inhibition or deletion of hepatocyte β_1_ integrin severely impairs liver regeneration (36, 37). Hepatocytes exhibit FAK activation adjacent to stiff collagen tracts in fibrotic liver in vivo and when cultured on top of stiff matrix in vitro (38), indicating that hepatocytes are mechanically responsive through FAK signaling. Moreover, data suggest that epithelial FAK signaling is protective in fibrotic disease because deletion of FAK in alveolar epithelial cells promotes apoptosis in response to TGF-β (39, 40) and results in greater lung injury and mortality in experimental models of lung fibrosis (39).

Given the potential benefits of FAK inhibition in treating malignant and/or fibrotic conditions, it is important to determine whether there may be adverse off-target effects on normal and protective epithelial functions. We hypothesized that liver epithelial FAK played important roles in maintaining hepatic homeostasis and in regulating response to fibrotic injury. We designed a series of experiments with liver epithelium–specific FAK-deficient mice to examine the hypothesis.

## Results

### RNA-Seq analysis suggests that FAK-deficient hepatocytes have decreased synthetic and proliferative functions and increased cell stress and lipid metabolism.

We bred FVB FAK^fl/fl^ mice (41) with albumin-Cre (Alb-Cre) mice (42) to generate liver epithelium–specific FAK-deficient mice (FAK^fl/fl^ Alb-Cre^+^ mice) and littermate controls (FAK^fl/fl^ Alb-Cre^–^). Because albumin is expressed in bipotential progenitor cells in the fetal liver, Alb-Cre–mediated gene deletion occurs within the fetal liver and manifests both in hepatocytes and cholangiocytes (43–47). We found that FAK^fl/fl^ Alb-Cre^+^ mice were born in the expected Mendelian ratios and had normal life spans (>1 year). FAK^fl/fl^ Alb-Cre^+^ mice aged 6–8 weeks old had liver FAK protein expression less than 15% of WT levels in littermate controls (Supplemental Figure 1; supplemental material available online with this article; https://doi.org/10.1172/jci.insight.141217DS1) and exhibited normal liver architecture histologically and normal serum liver function tests (Supplemental Figure 2).

**Figure 1:**
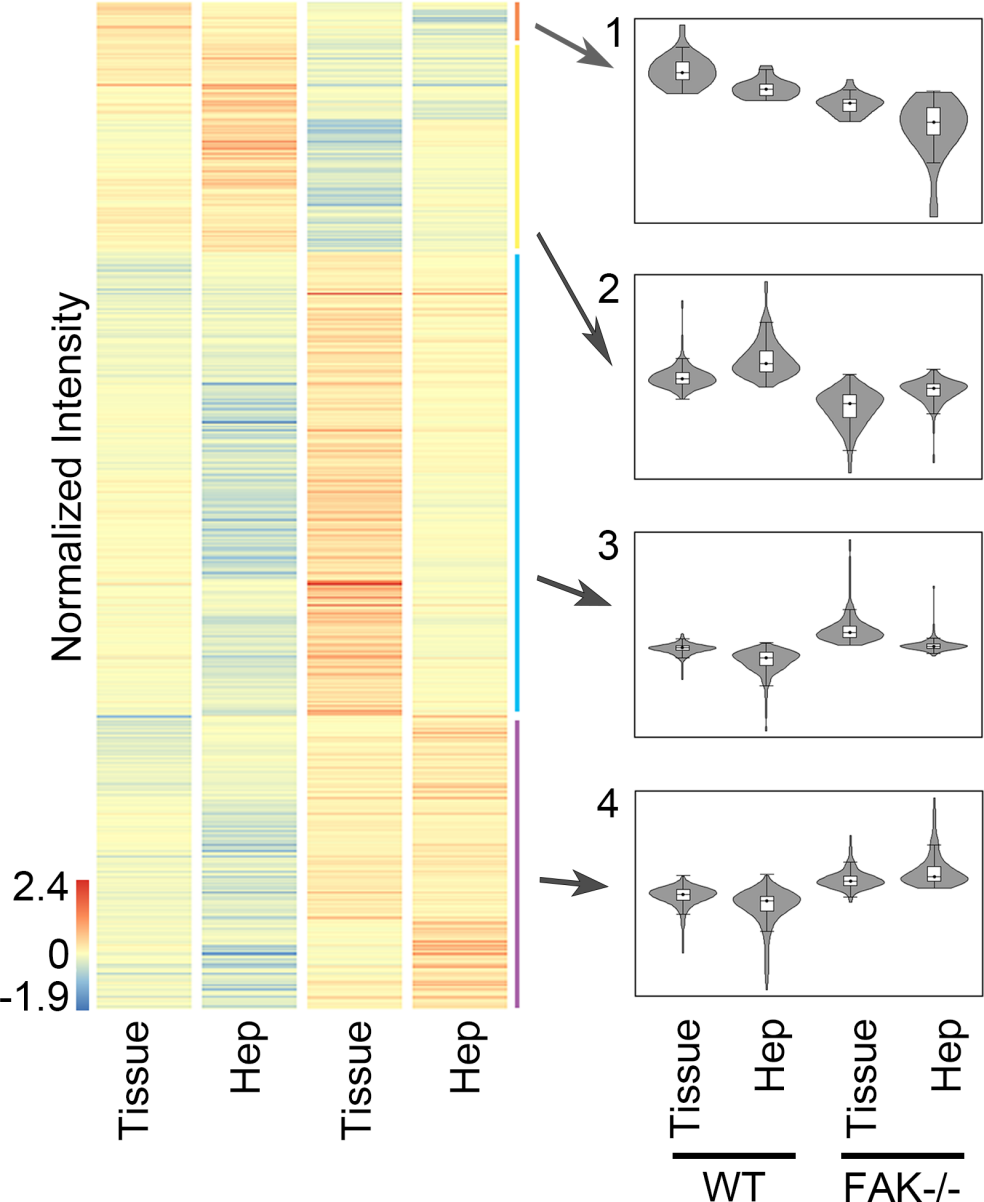
Whole-liver tissue or isolated hepatocytes from adult 6- to 8-week-old FAK^fl/fl^ Alb-Cre^+^ mice (FAK^–/–^) or FAK^fl/fl^ Alb-Cre^–^ littermate controls (WT) were analyzed by RNA-Seq. Heatmap shows hierarchical clustering of 782 significantly differentially expressed genes. Each column represents the averaged normalized expression of 4 samples (*n* = 4). Four major clusters emerged based on expression comparison to WT.

To determine whether liver epithelial FAK deletion induced cellular changes not readily detectable by gross phenotypic characterization, we performed RNA-Seq analysis on whole-liver tissue of 6- to 8-week-old FAK^fl/fl^ Alb-Cre^+^ mice and control FAK^fl/fl^ Alb-Cre^–^ littermates. We also performed RNA-Seq on isolated hepatocytes to distinguish hepatocyte-specific versus non–parenchymal cell–specific gene expression effects. Differential gene expression analysis of whole-liver tissue or isolated hepatocytes from FAK^fl/fl^ Alb-Cre^+^ compared with FAK^fl/fl^ Alb-Cre^–^ mice revealed 782 genes, which were hierarchically classified into 4 clusters based on expression pattern (Figure 1). Clusters 1 and 2 represented genes that were downregulated in FAK^fl/fl^ Alb-Cre^+^ liver and hepatocytes, whereas clusters 3 and 4 contained genes that were upregulated in FAK^fl/fl^ Alb-Cre^+^ liver and hepatocytes.

**Figure 2:**
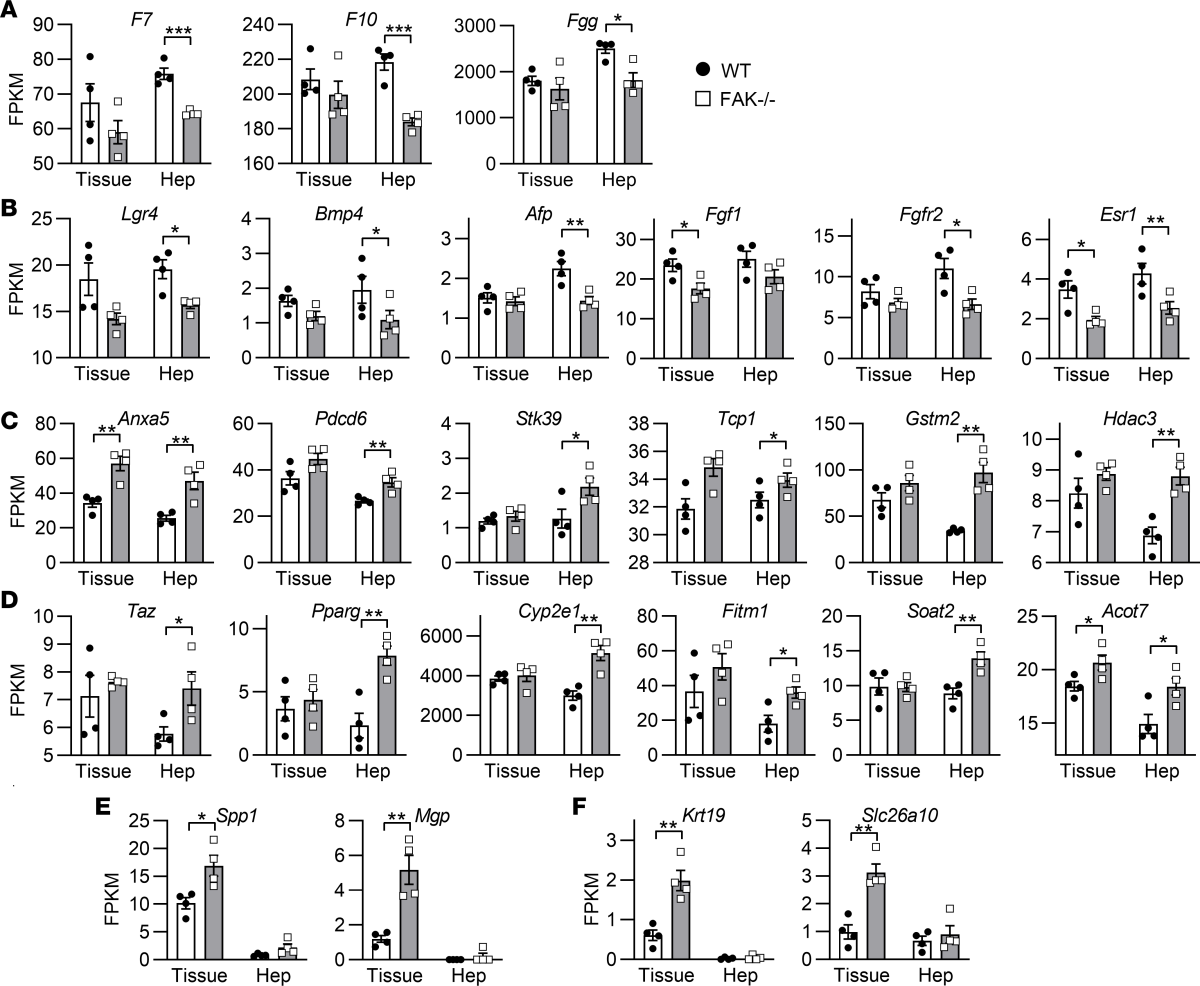
GO analysis of RNA-Seq revealed numerous gene categories that are differentially regulated in whole-liver tissue (Tissue) and isolated hepatocytes (Hep) of adult 6- to 8-week-old FAK^fl/fl^ Alb-Cre^+^ mice (FAK^–/–^) compared with FAK^fl/fl^ Alb-Cre^–^ littermate controls (WT). Expression of representative genes are shown for the GO terms (**A**) coagulation, (**B**) epithelial cell proliferation, (**C**) response to stress, and (**D**) lipid metabolism. Some genes are predominantly expressed in whole-liver tissue and not hepatocytes, indicating a non–parenchymal cell source, and include (**E**) extracellular matrix and (**F**) biliary-specific genes. Sample size *n* = 4 per group; *P < 0.05, **P < 0.01, and ***P < 0.001 by Student’s 2-tailed *t* test. Data represent individual data points and mean ± SEM. FPKM, fragments per kilobase of transcript per million base pairs.

Gene Ontology (GO) analysis showed that coagulation factors (Figure 2A), which represented a major class of serum proteins produced by hepatocytes, and cell proliferation genes (Figure 2B) were downregulated in FAK^fl/fl^ Alb-Cre^+^ samples. Conversely, genes associated with cell stress (Figure 2C) and lipid metabolism (Figure 2D) were upregulated in FAK^fl/fl^ Alb-Cre^+^ samples. Many genes within these functional categories were up- or downregulated more markedly in isolated hepatocytes than in whole-liver tissue, suggesting that these gene expression changes were hepatocyte specific. Several differentially regulated genes were nearly undetectable in isolated hepatocytes and showed differences only in whole-liver tissue, suggesting a non–parenchymal cell source. These included upregulation of extracellular matrix (Figure 2E) and biliary cell–specific genes (Figure 2F) in FAK^fl/fl^ Alb-Cre^+^ whole-liver tissues as compared with controls. These results suggest that liver epithelial FAK deficiency reduces hepatocyte-specific synthetic and proliferative functions, while increasing hepatocyte cell stress and lipid metabolism, and inducing activation of extracellular matrix and biliary cells within the liver.

### Liver epithelial FAK-deficient mice show greater signs of liver injury and steatosis with increased age as compared with age-matched controls.

**Figure 3:**
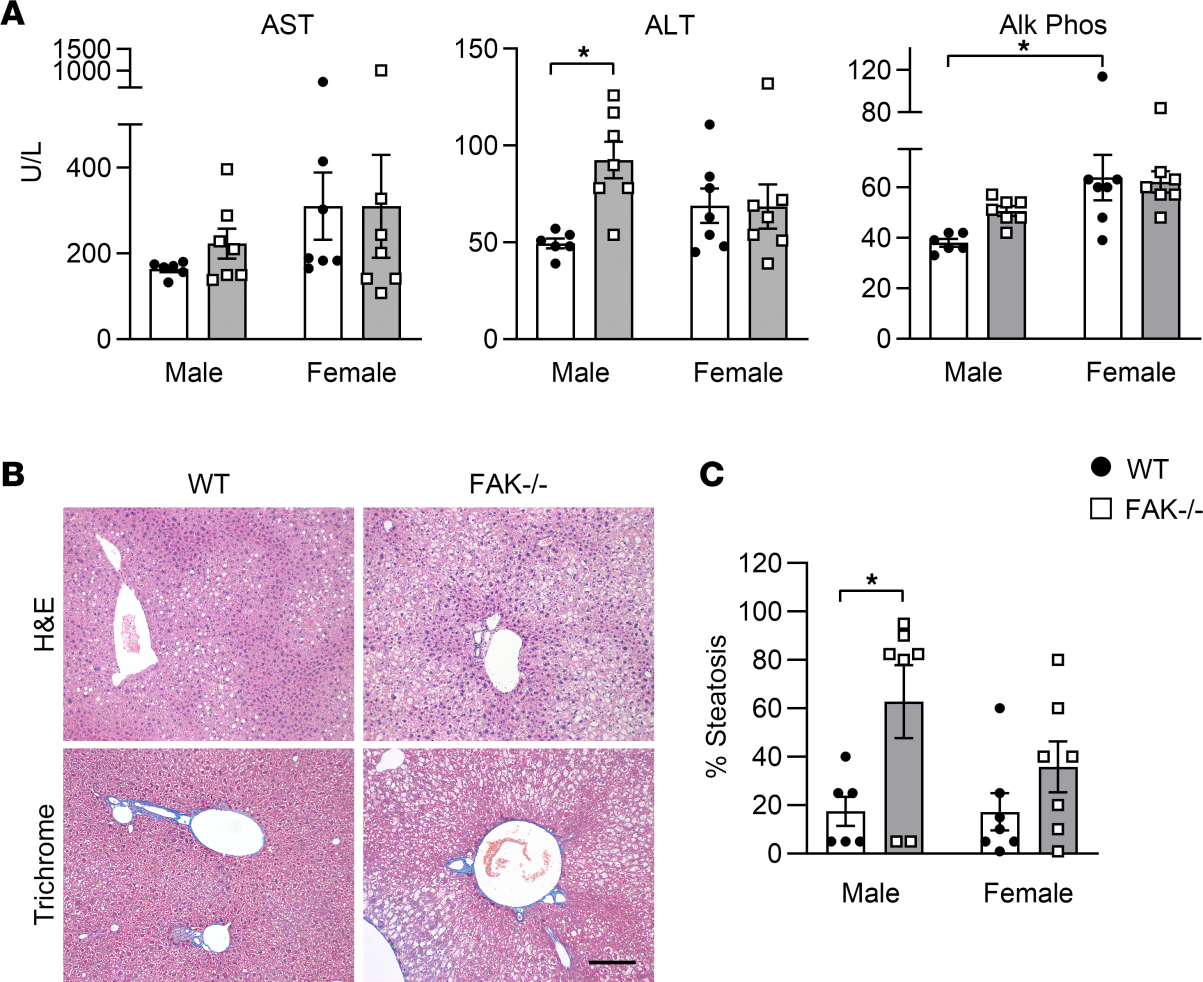
Male and female FAK^fl/fl^ Alb-Cre^+^ mice (FAK^–/–^) or FAK^fl/fl^ Alb-Cre^–^ littermate controls (WT) were aged to 6 months. (**A**) Serum liver function tests: aspartate transaminase (AST), alanine transaminase (ALT), and alkaline phosphatase (Alk Phos). (**B**) Representative liver H&E and trichrome histology of 6-month-old male WT and FAK^–/–^ mice. Scale bar: 200 μm. (**C**) The degree of steatosis as scored by a liver pathologist. For **A** and **C**, sample sizes are WT male *n* = 6, WT female *n* = 7, FAK^–/–^ male *n* = 7, and FAK^–/–^ female *n* = 7. Two-way ANOVA was performed with genotype (WT vs. FAK^–/–^) and sex (male vs. female) as independent variables; *P < 0.05 by post hoc Tukey’s honestly significant difference test. Data represent individual data points and mean ± SEM.

To determine whether gene expression indicating increased cell stress and lipid metabolism in FAK-deficient hepatocytes manifested phenotypically with time, we analyzed the serum liver function tests and liver histology of FAK^fl/fl^ Alb-Cre^+^ mice and FAK^fl/fl^ Alb-Cre^–^ littermate controls less than 40 g at 6 months of age. We found that liver epithelial FAK deletion induced liver injury and steatosis and that the effect differed between male and female mice. Two-way ANOVA indicated significant interaction (*P* = 0.025) between the effects of genotype (WT vs. FAK^–/–^) and sex (male vs. female) on serum alanine transaminase (ALT) levels (Figure 3A). Simple main effect analysis showed that FAK^fl/fl^ Alb-Cre^+^ mice had significantly higher ALT than WT littermates (*P* = 0.028), indicating greater hepatocellular injury. However, higher ALT was observed only in male FAK^fl/fl^ Alb-Cre^+^ compared with male FAK^fl/fl^ Alb-Cre^–^ mice; female FAK^fl/fl^ Alb-Cre^+^ and FAK^fl/fl^ Alb-Cre^–^ mice showed no difference. For alkaline phosphatase, sex was the main source of variation, with female mice showing higher levels than male mice (*P* = 0.002). These results suggest that absence of liver epithelial FAK signaling induces liver injury with time and that manifestations are sexually dimorphic.

Histologic analysis demonstrated increased steatosis, without evidence of steatohepatitis or fibrosis, in the livers of 6-month-old FAK^fl/fl^ Alb-Cre^+^ mice compared with age-matched controls (Figure 3B). A liver pathologist scored the severity of steatosis, and 2-way ANOVA indicated that FAK genotype was a significant source of variation (*P* = 0.007). Post hoc analysis demonstrated that male FAK^fl/fl^ Alb-Cre^+^ mice had significantly more steatosis than male controls, and female FAK^fl/fl^ Alb-Cre^+^ mice showed a similar trend compared with female controls (Figure 3C). These results show that increased expression of cell stress and lipid metabolism genes in FAK^fl/fl^ Alb-Cre^+^ mice eventually correlates with development of liver injury and steatosis as mice age.

### Liver epithelial FAK-deficient mice had significantly higher early mortality after bile duct ligation as compared with controls.

**Figure 4:**
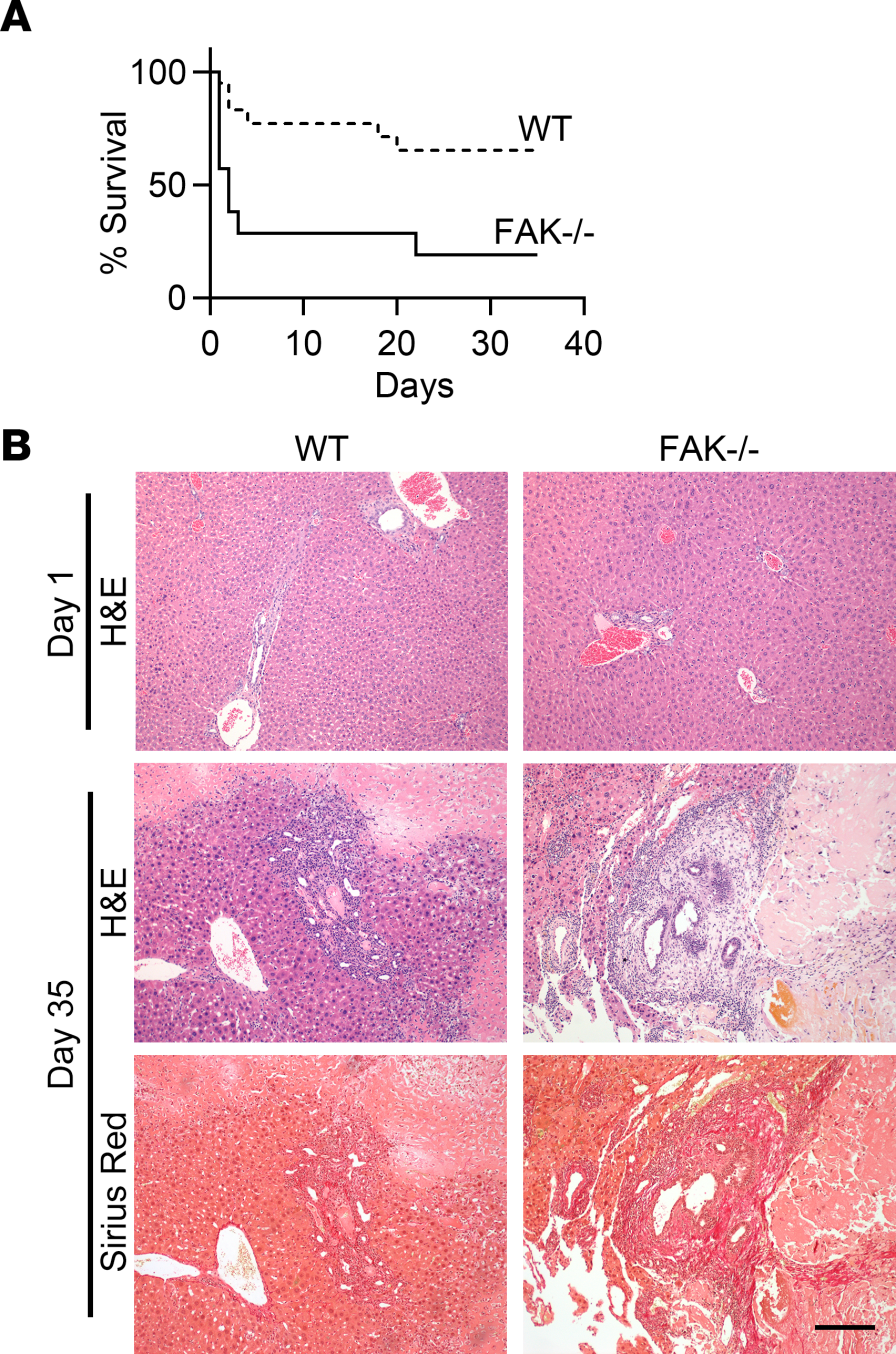
Male and female FAK^fl/fl^ Alb-Cre^+^ mice (FAK^–/–^) or FAK^fl/fl^ Alb-Cre^–^ littermate controls (WT) were subjected to bile duct ligation. (**A**) Kaplan-Meier survival analysis indicated significantly higher early mortality in FAK^–/–^ mice (male *n* = 8, female *n* = 6) compared with WT (male *n* = 12, female *n* = 8) after bile duct ligation, *P* < 0.01 by log-rank test. (**B**) Representative liver histology of FAK^–/–^ mice that died 1 day after bile duct ligation or survived until day 35. WT controls were sacrificed at the same time points for comparison. Scale bar: 200 μm.

Because liver epithelial FAK deficiency led to gradual hepatocellular damage with aging, we hypothesized that absence of epithelial FAK signaling would also impair adaptive responses to acute liver injury. Bile duct ligation is a surgical model of obstructive cholestasis that leads to severe liver fibrosis in 4 weeks (48). We performed bile duct ligation on male and female FAK^fl/fl^ Alb-Cre^+^ or FAK^fl/fl^ Alb-Cre^–^ mice between the ages of 6 and 8 weeks. Whereas the majority of FAK^fl/fl^ Alb-Cre^–^ control mice survived the immediate postoperative period, the vast majority of FAK^fl/fl^ Alb-Cre^+^ mice died within the first few days after the procedure (Figure 4A). Eleven out of 20 FAK^fl/fl^ Alb-Cre^–^ mice (55%) survived up to the end of the 5-week experimental period and developed fibrotic liver disease consistent with the model. In contrast, the median survival of FAK^fl/fl^ Alb-Cre^+^ mice after bile duct ligation was only 2 days, and only 2 out of 14 mice (14%) remained alive at 5 weeks. Survival rates did not differ between male and female mice. Liver histology of FAK^fl/fl^ Alb-Cre^+^ mice that died 1 day after bile duct ligation did not show obvious signs of liver parenchymal disruption or damage (Figure 4B). FAK^fl/fl^ Alb-Cre^+^ mice that survived until week 5 showed similar patterns of liver inflammation, parenchymal necrosis, and fibrosis as compared with FAK^fl/fl^ Alb-Cre^–^ controls. Nevertheless, these results suggest that liver epithelial cell FAK expression is critical for the immediate adaptive response to acute obstructive cholestatic injury.

### Liver epithelial FAK-deficient mice develop more severe liver injury and fibrosis in response to 3,5-diethoxycarbonyl-1,4-dihydrocollidine as compared with controls.

**Figure 5:**
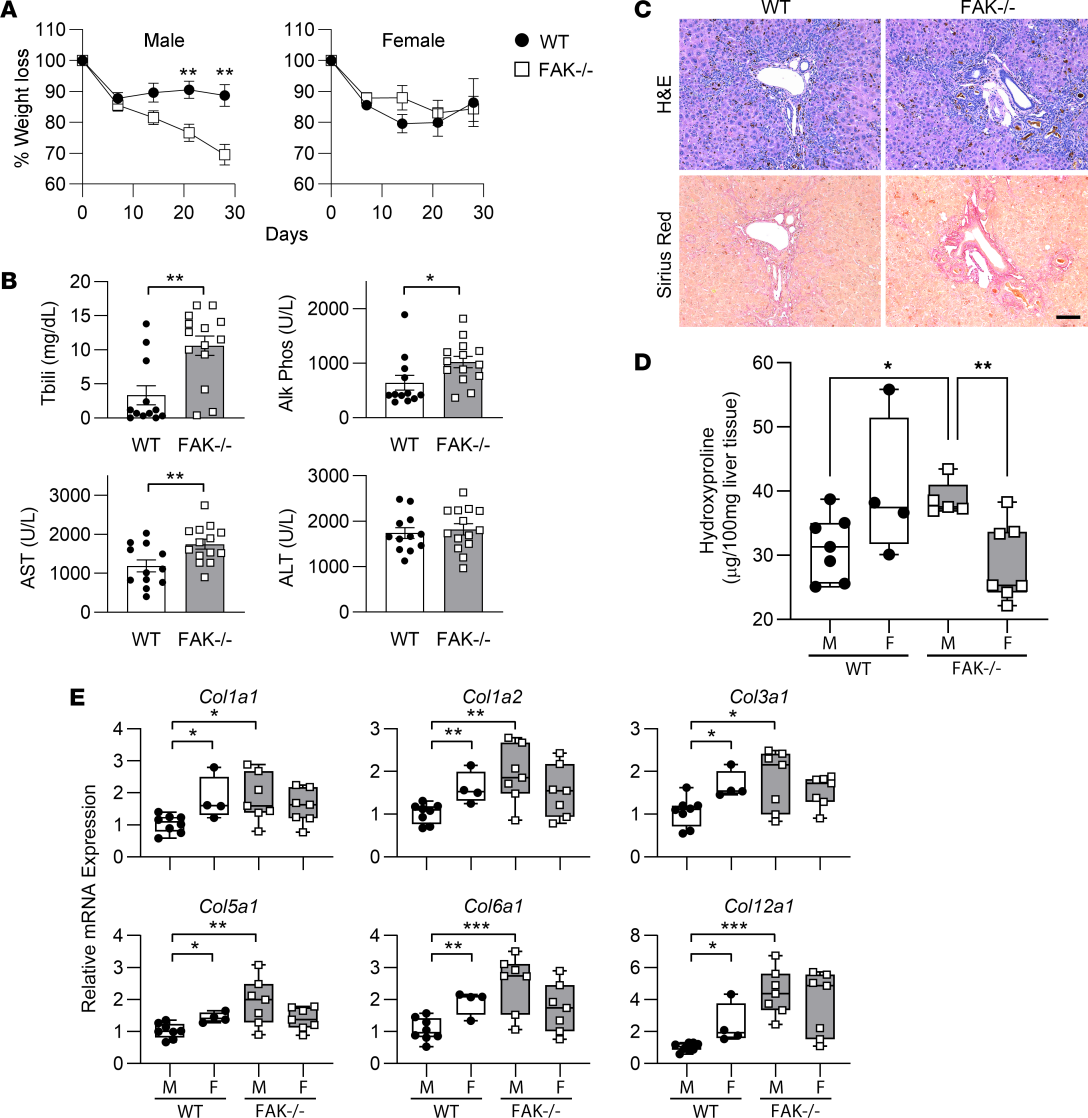
Male and female FAK^fl/fl^ Alb-Cre^+^ mice (FAK^–/–^) or FAK^fl/fl^ Alb-Cre^–^ littermate controls (WT) were given 0.1% DDC diet to induce liver fibrosis. (**A**) Male FAK^–/–^ mice (*n* = 7) lost significantly more weight than sex-matched controls (*n* = 12). Linear regression analysis showed that slopes of the 2 lines for male mice were significantly different (*P* < 0.05). Percentage weight loss in male mice was significantly different comparing WT and FAK^–/–^ by Student’s 2-tailed *t* test at days 21 and 28, **P < 0.01. Weight loss was not significantly different between female WT (*n* = 4) and FAK^–/–^ (*n* = 8). Data represent mean ± SEM. (**B**) Serum liver function tests for WT (male *n* = 8, female *n* = 4) and FAK^–/–^ mice (male *n* = 7, female *n* = 7). Data show individual data points and mean ± SEM. Tbili, total bilirubin. (**C**) Representative liver histology. Scale bar: 100 μm. (**D**) Liver hydroxyproline content of WT male (M; *n* = 7) and female (F; *n* = 4) compared with FAK^–/–^ male (*n* = 5) and female (*n* = 7) mice. (**E**) mRNA expression of various collagen species as determined by qRT-PCR in WT (M, *n* = 8; F, *n* = 4) and FAK^–/–^ (M, *n* = 7; F, *n* = 7) mice. Two-way ANOVA showed significant interaction between FAK genotype and sex (*P* < 0.05) for (**D**) and every collagen species except *Col12a1* in **E**; genotype contributed significantly to the overall variation for *Col5a1*, *Col6a1*, and *Col12a1* (*P* < 0.05). Groups were also compared by Student’s 2-tailed *t* test in **B**, **D**, and **E**, *P < 0.05, **P < 0.01, and ***P < 0.001. Box plots show individual data points, median, interquartile range, and range.

Bile duct ligation caused rapid early mortality in FAK^fl/fl^ Alb-Cre^+^ mice, and very few mice survived long enough to develop fibrosis. Therefore, to determine the effect of absent liver epithelial FAK signaling on fibrogenesis, we turned to the 0.1% 3,5-diethoxycarbonyl-1,4-dihydrocollidine (DDC) diet model that induces more gradual obstructive cholestasis and biliary fibrosis (49). All FAK^fl/fl^ Alb-Cre^–^ and FAK^fl/fl^ Alb-Cre^+^ mice on the DDC diet survived until the 4-week endpoint. Male FAK^fl/fl^ Alb-Cre^+^ mice lost significantly more weight than male FAK^fl/fl^ Alb-Cre^–^ controls, whereas weight loss did not differ between female FAK^fl/fl^ Alb-Cre^+^ and FAK^fl/fl^ Alb-Cre^–^ mice (Figure 5A). Compared with their littermate controls, both male and female FAK^fl/fl^ Alb-Cre^+^ mice demonstrated increased cholestasis, as indicated by serum total bilirubin and alkaline phosphatase, and hepatocellular injury, as indicated by serum AST (Figure 5B). Histologic analysis showed similar periportal inflammation, ductular reaction, and fibrosis between the 2 groups (Figure 5C). Quantitation of hydroxyproline to determine collagen content indicated that the livers of male FAK^fl/fl^ Alb-Cre^+^ mice had significantly more collagen, and thus more fibrosis, than those of male littermate controls (Figure 5D). Male FAK^fl/fl^ Alb-Cre^+^ mice also showed significantly more hydroxyproline than female FAK^fl/fl^ Alb-Cre^+^ mice. Using quantitative real-time reverse transcription PCR (qRT-PCR), we further analyzed the mRNA expression of fibrillar (*Col1a1*, *Col1a2*, *Col3a1*), microfibrillar (*Col6a1*), and fibril-associated (*Col12a1*) collagen species that are elevated in fibrotic liver diseases (50). We found that female WT FAK^fl/fl^ Alb-Cre^–^ mice showed higher expression of these collagen genes than male WT FAK^fl/fl^ Alb-Cre^–^ mice, suggesting that female mice developed more severe fibrosis compared with male mice at baseline. Importantly, the livers of male FAK^fl/fl^ Alb-Cre^+^ mice showed greater mRNA expression of all of these collagen species than did livers of male WT littermates (Figure 5E). These results indicate that liver epithelial FAK deficiency leads to increased DDC-induced liver fibrosis in male mice.

### Hepatocyte-specific FAK deletion results in greater expression of fibrotic collagen genes in response to DDC-induced fibrosis as compared with controls.

Studies suggest that FAK^fl/fl^ Alb-Cre^+^ mice delete FAK both from hepatocytes and cholangiocytes at the fetal liver stage (43–47). To determine the effect of absent hepatocyte FAK signaling immediately before profibrotic liver injury, we injected FAK^fl/fl^ mice with adeno-associated virus serotype 8–thyroxine binding globulin–Cre (AAV8-TBG-Cre) vector to induce FAK deletion only in hepatocytes (51). Control FAK^fl/fl^ mice were given AAV8-TBG-Null that did not express the Cre recombinase. DDC diet was initiated 2 weeks after viral vector administration, when liver FAK expression in AAV8-TBG-Cre–treated mice was reduced to less than 15% of WT control levels (Supplemental Figure 3).

**Figure 6:**
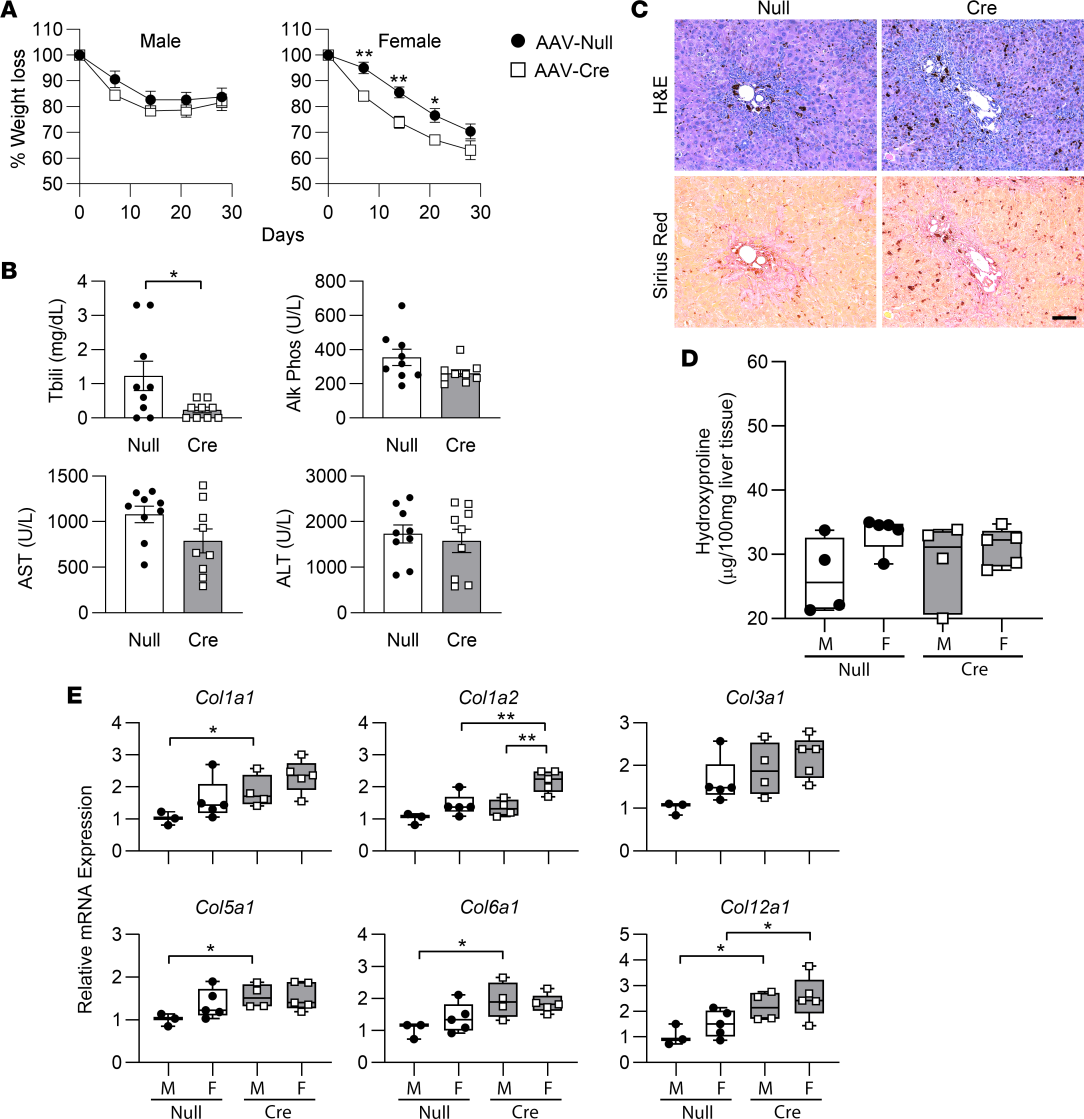
Male and female FAK^fl/fl^ mice were treated with AAV8-TBG-Cre (AAV-Cre) or AAV8-TBG-Null (AAV-Null) and then given 0.1% DDC diet to induce liver fibrosis. (**A**) Weight changes were not significantly different between male AAV-Null (*n* = 7) and AAV-Cre (*n* = 7) mice. Percentage weight loss in female mice was significantly different between AAV-Null (*n* = 5) and AAV-Cre (*n* = 5) at days 7, 14, and 21 by Student’s 2-tailed *t* test (*P < 0.05, **P < 0.01). Linear regression analysis showed that elevations of the AAV-Null and AAV-Cre female weight loss lines were significantly different (*P* < 0.0001). Data represent mean ± SEM. (**B**) Serum liver function tests for AAV-Null (male *n* = 4, female *n* = 5) and AAV-Cre mice (male *n* = 4, female *n* = 5). Data show individual data points and mean ± SEM. (**C**) Representative liver histology. Scale bar: 100 μm. (**D**) Liver hydroxyproline content of AAV-Null male (M; *n* = 4) and female (F; *n* = 5) compared with AAV-Cre male (*n* = 4) and female (*n* = 5) mice. (**E**) mRNA expression of various collagen species as determined by qRT-PCR in AAV-Null (M, *n* = 4; F, *n* = 5) and AAV-Cre (M, *n* = 4; F, *n* = 5) mice. For all collagen genes, 2-way ANOVA showed no significant interaction between AAV genotype and sex, whereas genotype contributed significantly to the overall variation (*P* < 0.05). For **B** and **E**, *P < 0.05 and **P < 0.01 by Student’s 2-tailed *t* test. Box plots show individual data points, median, interquartile range, and range.

In contrast to FAK^fl/fl^ Alb-Cre^+^ mice, male FAK^fl/fl^ mice injected with AAV8-TBG-Cre did not have greater weight loss than male mice treated with AAV8-TBG-Null (Figure 6A). Female FAK^fl/fl^ mice treated with AAV8-TBG-Cre initially lost weight more rapidly than female controls, though weights became comparable later. Total bilirubin levels were slightly lower in AAV8-TBG-Cre–treated mice compared with controls; alkaline phosphatase, AST, and ALT did not show significant differences (Figure 6B). Weight and serum liver function tests of mice treated with Null- and Cre-virus did not significantly change when DDC diet was extended to 8 weeks as compared with 4 weeks (data not shown). FAK^fl/fl^ mice treated with Null- and Cre-virus demonstrated similar levels of periportal inflammation, ductular reaction, and fibrosis (Figure 6C). Although no differences in liver collagen were detected by hydroxyproline assay (Figure 6D), AAV8-TBG-Cre–treated mice showed greater mRNA expression of fibrotic collagen genes as compared with controls (Figure 6E). Interestingly, in contrast to FAK^fl/fl^ Alb-Cre^+^ mice in which fibrotic collagen mRNA expression was increased only in males, AAV8-TBG-Cre–mediated FAK deletion led to increased collagen mRNA expression in both sexes. These results indicate that AAV-mediated hepatocyte FAK deletion produced less severe DDC-induced liver injury and fibrosis than Alb-Cre–mediated liver epithelial FAK deletion, but both methods of FAK deletion led to increased expression of fibrotic collagens.

### RNA-Seq analysis shows upregulation of several fibrosis regulatory pathways in FAK^fl/fl^ Alb-Cre^+^ mice and FAK^fl/fl^ mice treated with AAV8-TBG-Cre, compared with their respective controls.

**Figure 7:**
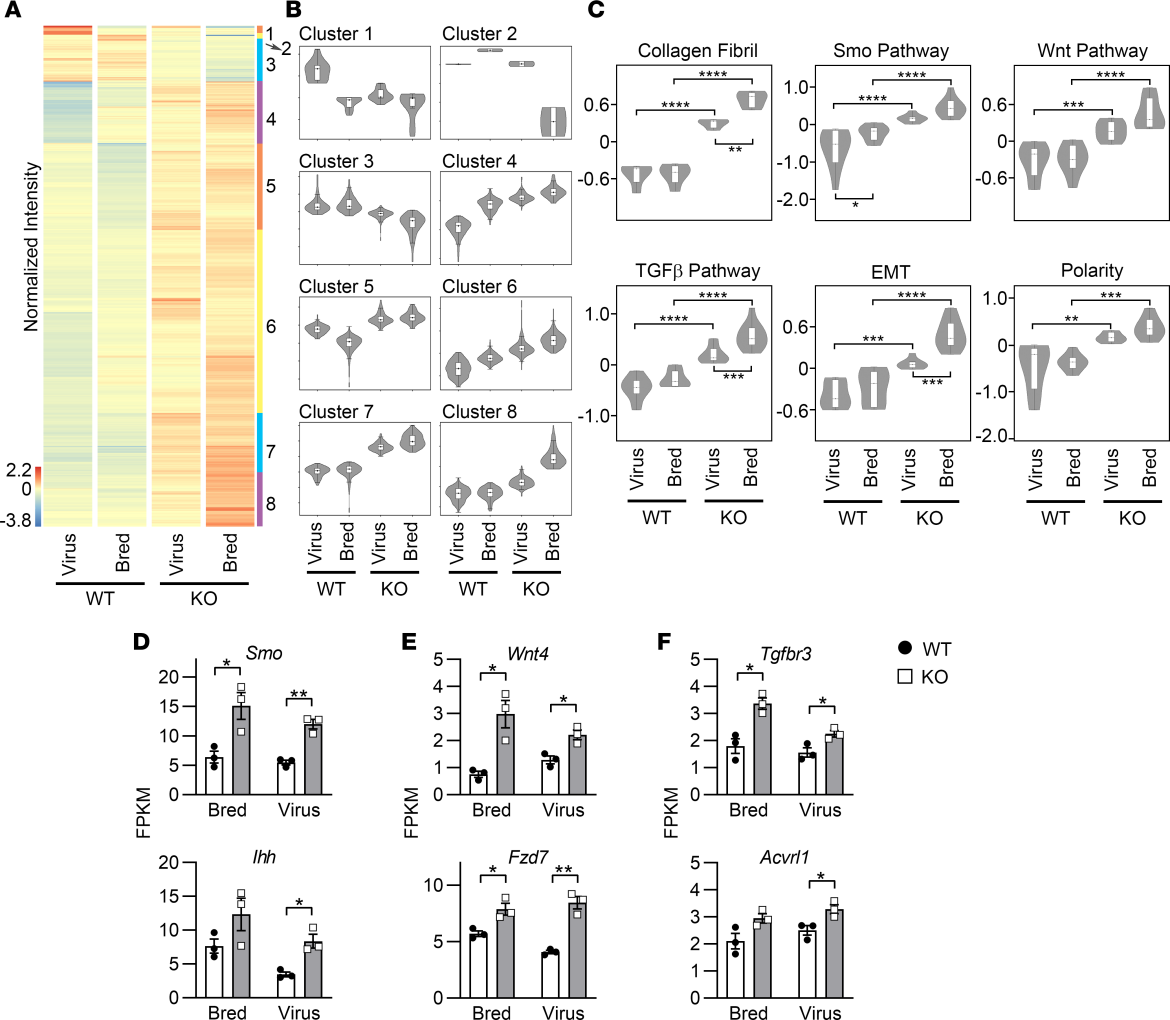
FAK^fl/fl^ mice treated with AAV8-TBG-Null (Virus WT) or AAV8-TBG-Cre (Virus KO), as well as FAK^fl/fl^ Alb-Cre^–^ (Bred WT) and FAK^fl/fl^ Alb-Cre^+^ (Bred KO) mice, were given 0.1% DDC diet to induce liver fibrosis. Whole-liver tissue from these mice was analyzed by RNA-Seq. (**A**) Heatmap shows hierarchical clustering of 896 significantly differentially expressed genes. Each column represents the averaged normalized expression of 3 samples (*n* = 3). (**B**) There were 8 gene clusters based upon expression patterns across the 4 groups. Cluster 1 contained 16 genes; cluster 2, 2 genes; cluster 3, 82 genes; cluster 4, 110 genes; cluster 5, 156 genes; cluster 6, 327 genes; cluster 7, 110 genes; and cluster 8, 93 genes. (**C**) GO analysis identified several processes and pathways that were upregulated in KO mice in both Virus and Bred methods of FAK deletion. Two-way ANOVA showed that there was significant interaction between the method of FAK deletion (Virus vs. Bred) and the genotype (WT vs. KO) for differentially regulated genes in the collagen fibril and epithelial-mesenchymal transition (EMT) categories (*P* < 0.05). The method of FAK deletion was a significant source of variation for collagen fibril, Smo, TGF-β, and EMT pathways (*P* < 0.01). For all 6 categories, genotype was a significant source of variation (*P* < 0.0001). Post hoc Tukey’s honestly significant difference test between the groups showed *P < 0.05, **P < 0.01, ***P < 0.001, and ****P < 0.0001. Expression levels of representative genes in the (**D**) Smo, (**E**) Wnt, and (**F**) TGF-β pathways were compared by Student’s 2-tailed *t* test, *P < 0.05 and **P < 0.01. For **D**–**F**, sample size was 3 per group (*n* = 3). Data represent individual data points and mean ± SEM.

We hypothesized that the absence of FAK signaling in hepatocytes activated profibrotic pathways, leading to increased transcription of fibrotic collagens. To identify these pathways, we performed RNA-Seq analysis on DDC-induced fibrotic whole-liver tissue from FAK^fl/fl^ Alb-Cre^+^ and FAK^fl/fl^ Alb-Cre^–^ mice (“Bred” method of FAK deletion), as well as FAK^fl/fl^ mice treated with AAV8-TBG-Cre or AAV8-TBG-Null (“Virus” method of FAK deletion). We defined FAK genotype (WT vs. KO) and method of FAK deletion (Bred vs. Virus) as independent variables in 2-way ANOVA, which revealed 896 significantly differentially regulated genes. We hierarchically classified these genes into 8 clusters based on their expression patterns across the 4 groups: Virus WT, Bred WT, Virus KO, and Bred KO (Figure 7, A and B). We were most interested in genes that were highly upregulated in KO mice induced by both Bred and Virus methods of deletion as compared with their respective WT controls (i.e., cluster 7). We hypothesized that genes with this expression profile represented pathways that were most robustly upregulated as a result of FAK deficiency in hepatocytes. Indeed, GO analysis of genes highly upregulated in both Bred and Virus methods of FAK deletion revealed several important fibrosis regulatory pathways (Figure 7C and Table 1). Confirming qRT-PCR results reported in Figure 5 and Figure 6, RNA-Seq analysis identified collagen fibril-forming genes as a significant functional category (*P* < 0.001) upregulated in both Bred and Virus KO mice. In addition, smoothened (Smo), Wnt, TGF-β, epithelial-mesenchymal transition, and polarity pathways were significantly upregulated both in Bred and Virus KO mice, as compared with their respective controls. Smo, Wnt, and TGF-β are master regulatory pathways of fibrogenesis (52–54). Epithelial-mesenchymal transition and changes in planar cell polarity are cellular processes implicated in the progression of fibrosis (55, 56). Consistent with FAK^fl/fl^ Alb-Cre^+^ mice developing more severe disease than FAK^fl/fl^ mice treated with AAV8-TBG-Cre, upregulation of many of these pathways was significantly greater in the Bred model than in the Virus model. We examined the expression of key gene members of the Smo, Wnt, and TGF-β pathways (Figure 7, D–F, respectively), and they were significantly more highly expressed in KO than in WT in both Bred and Virus models. These results indicate that multiple cross-regulatory profibrotic pathways are upregulated in the liver because of hepatocyte FAK deficiency in response to chronic fibrogenic injury.

### FAK-deficient hepatocytes show greater stiffness-dependent expression of Indian hedgehog; macrophages and biliary cells express elevated Smo in fibrotic hepatocyte-specific FAK-deficient liver.

**Figure 8:**
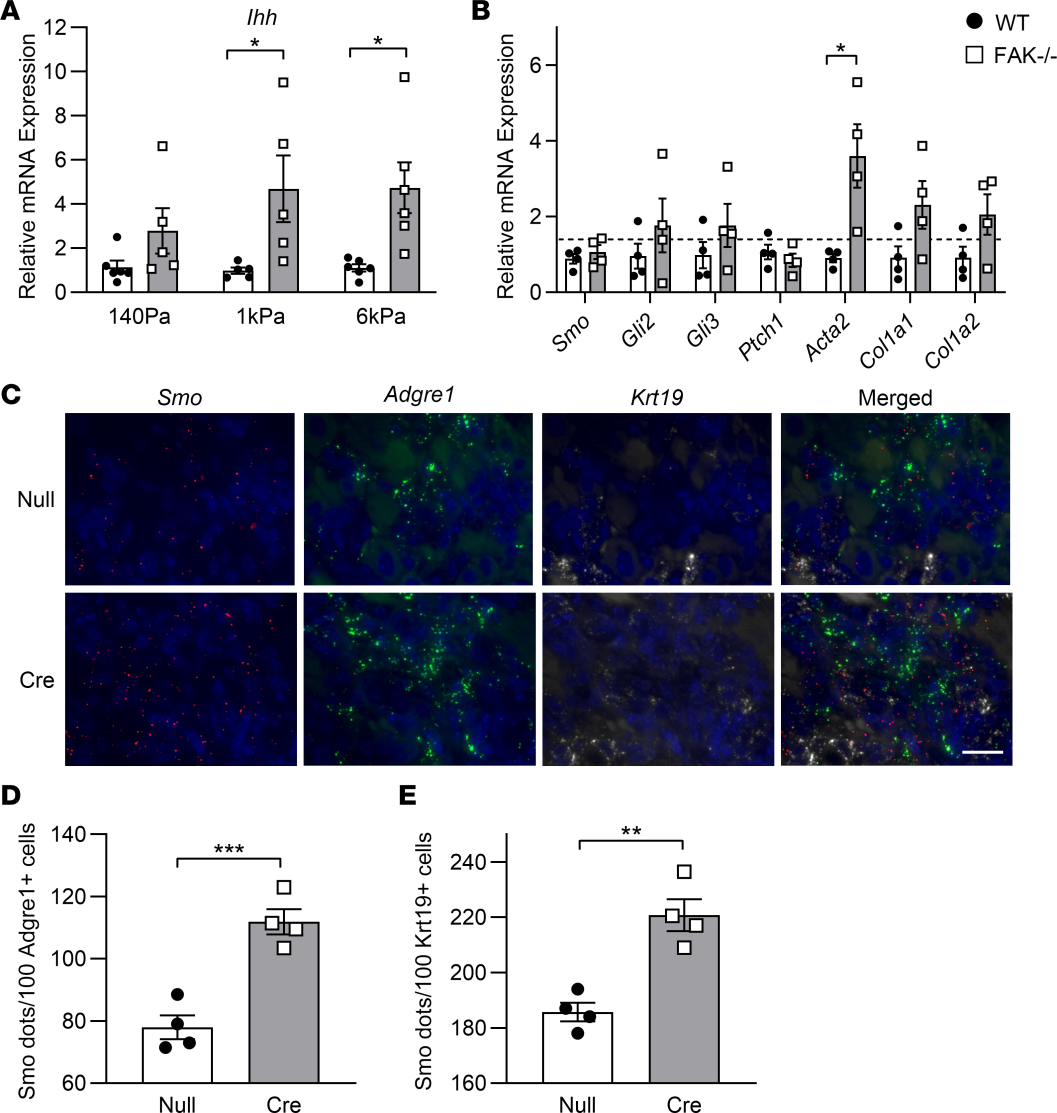
Hh/Smo pathway gene expression was determined in FAK-deficient hepatocytes and fibrotic hepatocyte-specific FAK-deficient liver. (**A**) Isolated hepatocytes from 6- to 8-week-old FAK^fl/fl^ Alb-Cre^–^ (WT) and FAK^fl/fl^ Alb-Cre^+^ (FAK^–/–^) mice on normal chow diet were cultured on collagen-coated polyacrylamide gels of 140 Pa, 1 kPa, or 6 kPa stiffness. *Ihh* mRNA expression was determined by qRT-PCR 24 hours later. Sample size *n* = 5–6 per group. (**B**) Stellate cells were isolated from WT and FAK^–/–^ mice with DDC-induced fibrosis. Expression of Hh/Smo pathway genes and stellate cell activation markers was determined by qRT-PCR. Sample size *n* = 4 per group. (**C**) Three-color multiplexed RNAscope analysis of *Smo*, *Adgre1*, and *Krt19* was performed on DDC-induced fibrotic liver tissues of FAK^fl/fl^ mice treated with AAV8-TBG-Null (Null) or AAV8-TBG-Cre (Cre). Images are representative of *n* = 4 per group. Scale bar: 20 μm. (**D**) Number of *Smo*^+^ dots that were colocalized with *Adgre1*^+^ macrophages. (**E**) Number of *Smo*^+^ dots that were colocalized with *Krt19*^+^ biliary cells. For **D** and **E**, 10 portal areas were analyzed per mouse, and at least 20 *Adgre1*^+^ or *Krt19*^+^ cells were analyzed per portal area; sample size was *n* = 4 per group. *P < 0.05, **P < 0.01, and ***P < 0.001 by Student’s 2-tailed *t* test. Data represent individual data points and mean ± SEM.

The hedgehog/Smo (Hh/Smo) pathway is a major profibrotic signaling pathway (53), and hepatocyte expression of Indian hedgehog (*Ihh*) has been shown to promote liver fibrosis (57). Our RNA-Seq data indicated that, compared with controls, hepatocyte-specific FAK-deficient fibrotic livers expressed significantly higher levels of both the ligand *Ihh* and the receptor *Smo* (Figure 7D). We hypothesized that profibrotic injury stimulated greater *Ihh* expression from FAK-deficient hepatocytes, leading to greater activation of *Smo*-expressing target cells and more severe fibrosis. To test this, we. isolated hepatocytes from the livers of 6- to 8-week-old FAK^fl/fl^ Alb-Cre^–^ and FAK^fl/fl^ Alb-Cre^+^ mice and cultured isolated hepatocytes on top of collagen-coated polyacrylamide gels of 140 Pa, 1 kPa, or 6 kPa stiffness. Previously, we had shown that 140 Pa was the matrix rigidity of normal liver, and 1–6 kPa was the stiffness of fibrotic liver matrix (38). WT hepatocytes isolated from FAK^fl/fl^ Alb-Cre^–^ mice showed uniformly low expression of *Ihh* on all 3 matrix stiffnesses. FAK-deficient hepatocytes demonstrated significantly higher levels of *Ihh* compared with WT when cultured on top of 1 kPa and 6 kPa gels (Figure 8A). Whole-liver tissue and freshly isolated hepatocytes from FAK^fl/fl^ Alb-Cre^–^ and FAK^fl/fl^ Alb-Cre^+^ mice did not show significant differences in *Ihh* expression (data not shown). These results indicate that absent FAK signaling in hepatocytes leads to increased *Ihh* expression in response to rigid fibrotic matrix.

Next, we wanted to identify the target cell(s) in fibrotic liver responsive to increased *Ihh* signals derived from FAK-deficient hepatocytes. Stellate cells are a major target of Hh signals and the producers of fibrotic matrix (58–63). We isolated stellate cells from FAK^fl/fl^ Alb-Cre^–^ and FAK^fl/fl^ Alb-Cre^+^ mice with DDC-induced liver fibrosis and determined fibrotic and Hh target gene expression (Figure 8B). Stellate cells from FAK^fl/fl^ Alb-Cre^+^ fibrotic livers showed higher activation (*Acta2*) and collagen expression (*Col1a1* and *Col1a2*) than in controls, consistent with the more clinically severe fibrosis that developed. However, expression of *Smo* and other Hh/Smo pathway genes (*Gli2*, *Gli3*, and *Ptch1*) was similar in stellate cells isolated from fibrotic FAK^fl/fl^ Alb-Cre^–^ and FAK^fl/fl^ Alb-Cre^+^ livers. These findings suggest that increased *Ihh* production by FAK-deficient hepatocytes promoted stellate cell activation and fibrillar collagen deposition through an indirect mechanism.

In order to identify the *Smo*^+^ *Ihh*-responsive cells, we performed multiplexed RNAscope analysis on DDC-induced fibrotic liver tissue from FAK^fl/fl^ mice treated with AAV8-TBG-Null or AAV8-TBG-Cre. We found that *Smo*^+^ cells were localized to periportal areas of inflammation and fibrosis (Figure 8C). Consistent with greater *Smo* expression as demonstrated by RNA-Seq (Figure 7), RNAscope showed that there were higher numbers of *Smo*^+^ dots in the periportal regions of AAV8-TBG-Cre–treated mice as compared with controls. *Smo*^+^ dots colocalized with cells that expressed *Adgre1* (also known as F4/80), which marked activated macrophages. In addition, *Smo*^+^ dots colocalized with cells that expressed cytokeratin 19 (*Krt19*), which marked biliary cells, proliferating ductular cells, and progenitor cells within ductular reactions. We quantified the numbers of *Smo*^+^ dots in *Adgre1*^+^ cells (Figure 8D) and *Krt19*^+^ cells (Figure 8E) and found that these cells in AAV8-TBG-Cre–treated mice expressed significantly more *Smo* than did controls. These results suggest that absence of hepatocyte FAK signaling induces greater hepatocyte *Ihh* expression, which activates Smo-dependent signaling in macrophages and biliary cells, leading to more severe fibrosis.

## Discussion

Our study shows that liver epithelium–specific deletion of FAK led to hepatocyte dysfunction and cell stress, which eventually manifested as liver injury as mice aged. Mice with liver epithelial FAK deficiency died rapidly when challenged with acute biliary obstruction and developed more severe fibrosis in response to chronic cholestatic injury. Absence of hepatocyte FAK induced upregulation of several profibrotic signaling pathways, including the Hh/Smo pathway. Moreover, FAK-deficient hepatocytes showed increased expression of *Ihh* that likely stimulated *Smo*^+^ macrophages and biliary cells to promote fibrogenesis. These findings indicate that FAK signaling has critical roles in the homeostatic function of hepatocytes and in regulating liver fibrosis progression.

Our results are consistent with previously published observations that FAK^fl/fl^ Alb-Cre^+^ mice do not develop obvious liver abnormalities at the typical adult age investigated in laboratory studies (i.e., 6–8 weeks old) (16). We showed that liver damage because of absent liver epithelial FAK signaling accumulated over time and was triggered by acute or chronic insults. Accordingly, our findings are also consistent with data indicating that integrin-mediated signals in hepatocytes have important homeostatic and protective functions. Prolonged hepatocyte β_1_ integrin knockdown distorts bile canaliculi morphology, induces liver dysfunction, and inhibits liver regeneration (35–37). Moreover, our results add to the body of evidence that epithelial FAK signaling has a regulatory role in response to organ injury and fibrosis. Alveolar epithelial FAK deletion induces greater epithelial apoptosis, lung injury, and mortality in experimental lung fibrosis (39, 40). Similarly, we showed that liver epithelial FAK deletion led to greater liver injury, fibrosis, and activation of profibrotic pathways in experimental liver fibrosis.

The 2 methods, Bred and Virus, that we used to induce liver epithelial FAK deletion yielded different results. Overall, in response to DDC, FAK deletion through the Bred method demonstrated greater liver injury, fibrosis, and activation of profibrotic pathways than the Virus method. There could be at least 2 nonmutually exclusive reasons for this finding. Our results indicated that the absence of hepatocyte FAK signaling caused increased cell stress that manifested as hepatocellular injury over time. Because the Bred method induced FAK deletion in the fetal liver, FAK^fl/fl^ Alb-Cre^+^ mice might have shown more marked liver injury and fibrosis because their livers were already under significant stress because of lifelong absence of FAK. In contrast, in the Virus method, FAK^fl/fl^ mice treated with AAV8-TBG-Cre developed FAK deficiency just before initiation of DDC-induced injury; the livers of these mice might have been relatively more robust and more able to withstand the profibrotic insult. Another consideration is that the Bred method deleted FAK both in hepatocytes and cholangiocytes (43–47), whereas the Virus method deleted FAK only in hepatocytes (51). It is possible that FAK plays an important role in cholangiocytes as well as in hepatocytes (64), and thus, the absence of FAK in both cell types induces more severe liver injury and fibrosis in FAK^fl/fl^ Alb-Cre^+^ mice. Importantly, our results show that AAV8-TBG-Cre–mediated FAK deletion was sufficient to induce greater fibrotic collagen expression and activation of profibrotic pathways, indicating that the absence of FAK in hepatocytes alone was enough to promote more severe liver fibrosis.

Our results showed that differences between male FAK^fl/fl^ Alb-Cre^+^ mice and male controls were more significant than between female FAK^fl/fl^ Alb-Cre^+^ mice and female controls. Six-month-old male FAK^fl/fl^ Alb-Cre^+^ mice demonstrated significantly increased serum ALT and steatosis (Figure 3), whereas female mice did not, indicating that sex modified the manifestations of liver epithelial FAK deficiency. Sexual dimorphism in liver function and diseases is well documented (65, 66). Sex-specific differences we observed in DDC-induced liver fibrosis may be at least partially attributable to differential cytochrome-mediated metabolism of DDC in male and female mice (67). Importantly, regulation of FAK signaling may be sexually dimorphic in ways both dependent and independent of sex hormones. Estrogen receptor activation in human ovarian cancer cells activates FAK (68). In mice, there is a female-specific vitronectin/FAK/IL-6 regulatory circuit that is independent of sex hormones (69). Vascular smooth muscle cells from female rats are more resistant to anoikis and exhibit increased FAK activation and survival by autophagy compared with cells from male rats (70). These observations, along with our results, suggest that sexually dimorphic effects of FAK signaling may be due to a variety of different mechanisms. Greater understanding of the sex-specific effects of FAK activation is of interest for future research and will provide crucial insight for the clinical translation of FAK-based therapies to female and male patients.

Finally, our results indicate that hepatocyte FAK deficiency activates Hh/Smo, a pleiotropic signaling pathway with key regulatory roles in embryonic development, cancer, and fibrosis (53, 71, 72). Hh/Smo signaling is activated in the course of fibrogenesis in multiple organs, including the liver, and inhibition of Hh/Smo reduces fibrosis progression (53). Whereas normal adult liver produces very little Hh ligand, injured hepatocytes are an important source of *Ihh* (57, 73, 74). Many cell types respond to Hh signals in the inflammatory fibrotic liver microenvironment, including stellate cells (58–63), macrophages (73, 75), cholangiocytes (76–78), and progenitor cells (59, 73, 74, 76). Our results suggest that FAK-deficient hepatocytes upregulate *Ihh* expression and stimulate Smo^+^ macrophages and bile ductular cells in periportal areas of inflammation. Other studies have shown that Hh-responsive macrophages and cholangiocytes secondarily activate stellate cells through paracrine cytokines/chemokines and thereby increase fibrotic collagen production (73, 75–78). Our data are consistent with a model in which FAK-deficient hepatocytes produce *Ihh* that stimulates Smo^+^ macrophages and biliary cells, which in turn stimulate stellate cells to increase production of fibrotic collagens. Although we showed that stellate cells in FAK-deficient fibrotic livers did not upregulate Smo and other Hh/Smo pathway genes, hepatocyte-derived *Ihh* could still activate stellate cells through noncanonical, Smo-independent, Gli-independent signaling (73). Future studies are needed to elucidate the precise mechanisms of how hepatocyte FAK signaling regulates Hh/Smo intercellular communication and subsequent fibrosis progression. Moreover, upregulated *Ihh* in FAK-deficient hepatocytes might represent a generalized response of injured hepatocytes (74), or alternatively, might be due to dysregulated mechano-sensing in the absence of FAK signaling, as suggested by our results that FAK-deficient hepatocytes expressed greater *Ihh* when cultured on matrices of higher rigidity. Compensatory and/or parallel mechano-sensing mechanisms, such as ILK, Src, Rho, and YAP/tafazzin (TAZ), might be stimulated in the absence of FAK and induce aberrant *Ihh* expression. Interestingly, TAZ has been shown to induce hepatocyte expression of *Ihh* in nonalcoholic steatohepatitis (57), and ILK has been shown to interact with Smo to mediate Hh signaling (79).

In conclusion, our findings show that liver epithelial FAK has important roles in maintaining liver homeostasis and in regulating liver fibrosis. Although these findings raise caution for the use of FAK inhibitors in treating malignant and fibrotic conditions, it is likely that oncogenic cells and myofibroblasts have different set points of FAK activity than normal epithelial cells. FAK inhibition may remain a promising strategy if specific cell types of interest can be targeted, while sparing normal epithelial cells. Additional studies to determine the safety, efficacy, and potential off-target effects of FAK inhibitor–based therapies are needed.

## Methods

### Mice.

Liver epithelium–specific FAK-deficient mice (FAK^fl/fl^ Alb-Cre^+^) and littermate controls (FAK^fl/fl^ Alb-Cre^–^) were generated by breeding FVB FAK^fl/fl^ mice (gift from Hilary E. Beggs, Calico, South San Francisco, California, USA) (41), in which the FAK gene was flanked by *loxP* sites, with Alb-Cre mice (gift from Derek LeRoith, Icahn School of Medicine at Mount Sinai, New York, New York, USA) that carry the Cre recombinase under the albumin promoter (42). Hepatocyte-specific FAK deletion was induced by tail vein injection of FAK^fl/fl^ mice with 2.5 × 10^11^ AAV8-TBG-Cre (gift from James M. Wilson, Perelman School of Medicine, University of Pennsylvania, Philadelphia, Pennsylvania, USA; Addgene viral prep 107787-AAV8; RRID: Addgene_107787). Littermate controls were injected with 2.5 × 10^11^ viral particles/mouse of control viral vector that did not express Cre recombinase (AAV8-TBG-Null). All experimental mice were cared for in accordance with the NIH *Guide for the Care and Use of Laboratory Animals* (National Academies Press, 2011).

### Liver fibrosis models.

Acute obstructive cholestasis was induced by bile duct ligation using standard described surgical techniques (80). Gradual cholestatic injury was induced by providing mice with 0.1% w/w DDC diet (TestDiet) ad libitum for 4–8 weeks (49).

### Serum and tissue analyses.

Blood and liver were collected from mice under inhaled isoflurane anesthesia. Fresh, unfrozen sera were analyzed by ADVIA Chemistry XPT System (Siemens) for AST, ALT, total bilirubin, and alkaline phosphatase. The entire liver was recovered and apportioned for histologic, RNA, protein, and total collagen content analyses. For histology, liver tissues were fixed in 10% buffered formalin, and the UCSF Liver Center Pathology and Imaging Core (San Francisco, California, USA) performed H&E and Sirius red staining using standard techniques. A liver pathologist qualitatively evaluated all histology samples for steatosis, inflammation, and fibrosis.

### RNA-Seq.

Total RNA was isolated using the RNeasy Plus Mini Kit (QIAGEN), and RNA quantity and integrity were measured by 2100 Bioanalyzer (Agilent Technologies). Library construction, high-throughput sequencing, quality control, and sequence alignment were performed by Novogene Inc. per the company’s standard procedures. Briefly, 1 μg of total RNA was used for the sample preparations. rRNA was removed by the Ribo-zero rRNA removal kit (Epicentre) and the residual RNA cleaned by ethanol precipitation. Sequencing libraries were generated by NEBNext Ultra Directional RNA Library Prep Kit for Illumina (New England Biolabs) following the manufacturer’s protocols. Library fragments were purified with AMPure XP system (Beckman Coulter) to obtain 250–300 bp cDNA fragments. Library quality was assessed by Agilent Technologies Bioanalyzer 2100, and high-throughput sequencing was performed on the Illumina HiSeq 4000 platform using HiSeq 3000/4000 SBS Kit (300 cycles) with 20 million reads per sample. Raw reads in FASTQ format were processed by removing the adaptor reads or poly-N and low-quality reads. Error rate distribution and Q20, Q30, and GC content of the cleaned data were calculated. Quality-controlled reads were mapped to the mouse reference genome (mm10) with STAR alignment software (81). Gene expression levels were analyzed according to FPKM reads sequenced. The MINSEQE-compliant data set is available through the National Center for Biotechnology Information’s Gene Expression Omnibus public repository (accession GSE157156).

### Total liver collagen quantification.

Total collagen content in liver tissues was determined by using a hydroxyproline assay kit (MilliporeSigma). Briefly, 100–150 mg tissue per liver sample was hydrolyzed at 110°C for 20 hours in 1 mL 6N HCl per 100 mg tissue. Hydroxyproline standards and 10 μL of hydrolyzed samples were added to 96-well flat-bottom plates and evaporated to dryness on a 60°C heat block. The sediment was dissolved in 100 μL of chloramine-T/oxidation buffer mixture for 10 minutes; then 100 μL of p-dimethylaminobenzaldehyde/perchloric acid/isopropanol mixture was added to each well and incubated at 60°C for 90 minutes. Samples were cooled to room temperature, with absorbance measured at 562 nm.

### Quantitative real-time reverse transcription PCR.

Total RNA was extracted using the RNeasy Plus Mini Kit (QIAGEN) according to the manufacturer’s protocol. RNA concentration was measured by the NanoDrop1000 spectrophotometer (Thermo Fisher Scientific) and RNA purity verified by 260/280 absorbance, which consistently ranged between 1.8 and 2.0. Reverse transcription was carried out with 100 ng of RNA using the High-Capacity cDNA Reverse Transcription Kit (Applied Biosystems, Thermo Fisher Scientific) following the manufacturer’s instructions. A total of 1 μL of the resulting cDNA was added to a final 10 μL mixture containing 5 μL of 2× SYBR Green PCR Master Mix (Life Technologies, Thermo Fisher Scientific) and 3 pmol oligonucleotide primers. qPCRs were carried out in a 7300 Real-Time PCR System (Applied Biosystems, Thermo Fisher Scientific) using the thermal profile 50°C for 2 minutes and 95°C for 10 minutes, followed by 40 amplification cycles consisting of 95°C for 15 seconds, 60°C for 30 seconds, and 72°C for 30 seconds. Samples were normalized to rRNA 18S internal standard. Relative quantification of gene expression was calculated by using the 2^ΔΔCt^ equation. Sequences of primers used in qRT-PCRs are listed in Supplemental Table 1.

### Primary mouse hepatocyte and stellate cell isolation.

Hepatocyte isolation was achieved through 2-step perfusion of the liver in situ using Liver Perfusion and Liver Digest Media (Thermo Fisher Scientific) and then density separation by 50% Percoll (GE Healthcare Life Sciences) gradient. Stellate cell isolation was performed following previously published protocols (82). Briefly, fibrotic livers were perfused in situ with 1.2 mg/mL pronase (Roche) and 0.25 U/mL collagenase (Crescent Chemical) and then digested in vitro with 0.5 mg/mL pronase, 0.1 U/mL collagenase, and 0.1 mg/mL DNase I (Roche) for 10 minutes in a 250 rpm shaker at 37°C. Stellate cells were separated by density gradient centrifugation using 12% Accudenz (Accurate Chemical) at 1380*g* for 17 minutes at 4°C.

### Collagen-conjugated polyacrylamide gels.

Circular, 22 mm, glass coverslips (VWR) were activated by 0.5% 3-aminopropyl trimethoxysilane and glutaraldehyde (MilliporeSigma). Different ratios of 40% acrylamide and 2% bis-acrylamide (Bio-Rad) were combined to generate 140 Pa, 1 kPa, and 6 kPa stiffness gels as calibrated previously (83). Polymerization was initiated by tetramethylethylenediamine (Bio-Rad) and potassium persulfate (MilliporeSigma). Then 0.01% bis-acrylamide, 0.002% di(trimethylolpropane) tetraacrylate (MilliporeSigma), 0.025% Irgacure (BASF Resins), 0.006% acrylic acid N-hydroxysuccinimide ester (MilliporeSigma), and 25% ethanol in 50 mM HEPES (pH 6.0) were applied, and gels were exposed to 245 nm ultraviolet light for 300 seconds for photoactivation. Gels were thoroughly washed in phosphate-buffered saline to remove excess reagent and incubated with 150 mg/mL rat tail collagen I (VWR) solution overnight at 4°C for conjugation. Before cells were plated, the gels were equilibrated in DMEM (Mediatech) at 37°C overnight. Primary hepatocytes were cultured at 50,000 cells/gel/well in 12-well plates (Eppendorf) in DMEM supplemented with 5% FBS (Hyclone), l-glutamine, antibiotics, insulin-transferrin-selenium, and HEPES (Mediatech).

### RNAscope in situ hybridization.

Multiplex RNAscope assay of mouse liver tissue was performed by using probes and reagents from Advanced Cell Diagnostics and following the manufacturer’s protocol. Briefly, 5 μm–thickness tissue sections were baked at 60°C for 1 hour, deparaffinized, and treated with hydrogen peroxide at room temperature for 10 minutes. Target retrieval was performed by putting slides into 100°C target retrieval reagent for 30 minutes, followed by protease treatment at 40°C for 30 minutes. Mouse *Smo* (318411-C1), *Adgre1* (460651-C2), and *Krt19* (402941-C3) probes were then hybridized at 40°C for 2 hours. Hybridization with preamplifier, amplifier, and fluorescent labeling probes was performed by using RNAscope Multiplex Fluorescent Detection Kit v2 (Advanced Cell Diagnostics) and opal520, opal570, and opal650 (Akoya Biosciences) at 40°C. Images were acquired using a Leica DM6B microscope with a 4.2 MP CMOS camera (Leica Microsystems). For quantification, 10 high-power field images were randomly taken per sample, and fluorescent *Smo* spots were counted from 20 randomly selected *Adgre1*^+^ or *Krt19*^+^ cells per image; i.e., Smo spots were counted in 200 randomly selected *Adgre1*^+^ and *Krt19*^+^ cell per sample.

Further information can be found in Supplemental Methods.

### Statistics.

RNA-Seq differential gene expression and clustering analyses using FPKM were performed with GeneSpring GX v14.9 (Agilent Technologies). We used moderated t-test with corrected *P* < 0.05 to determine differential gene expression between baseline WT and FAK^–/–^ samples (*n* = 8 per group; pooled whole-liver tissue *n* = 4; and isolated hepatocyte *n* = 4 for each genotype). For fibrotic liver samples, genotype (WT vs. KO) and method of FAK deletion (Bred vs. Virus) were assigned as independent variables in 2-way ANOVA with Benjamini-Hochberg multiple-testing correction (corrected *P* < 0.05 and *n* = 3 per group). Heatmap hierarchical clustering on normalized intensity values was performed on genes and conditions using Pearson centered or Euclidean similarity measure and Ward’s linkage. Violin plots show median, interquartile range, and range. GO analysis was performed with the GeneSpring software or GOrilla web-based tool (84) using *P* value threshold less than 0.001.

Additional statistical analyses, including survival analysis, log-rank test, regression analysis, 2-way ANOVA, and 2-tailed Student’s *t* tests, were performed with Prism v8.4.2 (GraphPad). Where appropriate, 2-way ANOVA was performed with genotype (WT vs. KO) and sex (male vs. female) as independent variables. Otherwise, 2-tailed Student’s *t* test was used to test significance. Box plots show individual data points, median, interquartile range, and range. Bar graphs show individual data points and mean, with error bars representing SEM.

### Study approval.

Animal studies were approved by UCSF IACUC (approval AN176137).

## Author contributions

YW designed and conducted experiments, analyzed data, and wrote the manuscript; TJL, VXZ, MLI, MAP, TKB, and MCY conducted experiments; WTC evaluated histology; TTC conceived the research, designed and conducted experiments, analyzed data, and wrote the manuscript.

## Supplementary Material

Supplemental data

## Figures and Tables

**Table 1:**
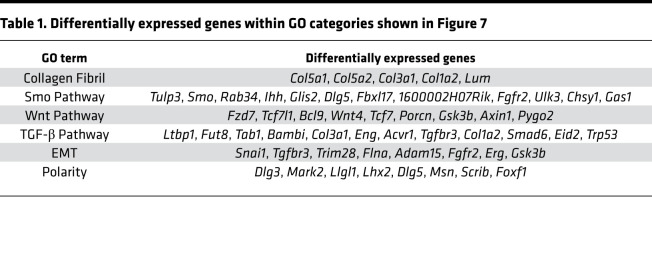
Differentially expressed genes within GO categories shown in Figure 7

## Version 1 09/10/2020

In-Press Preview

## Version 2 10/15/2020

Electronic publication
