# Supplementary material for: Liver epithelial focal adhesion kinase modulates fibrogenesis and hedgehog signaling
Source: JCI Insight. 2020 Oct 15;5(20):e141217. doi: 10.1172/jci.insight.141217 (PMC7605528; doi:10.1172/jci.insight.141217)
Supplement: Supplemental data [file jciinsight-5-141217-s062.pdf]

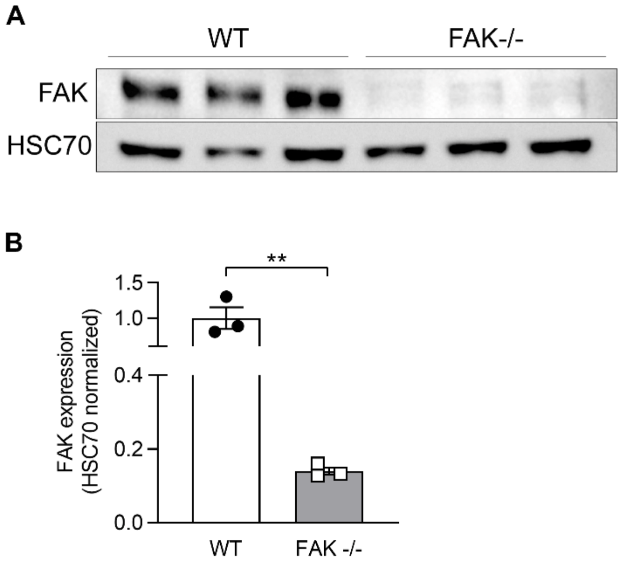

Supplemental Figure 1. There is efficient deletion of FAK protein expression in the liver of 6-8 week-old FAK$^{fl/fl}$;Alb-cre^{+} mice (FAK-/-), compared to FAK$^{fl/fl}$;Alb-cre^{-} littermate controls (WT). (**A**) Western blot shows FAK protein expression in whole liver tissue lysate of WT and FAK-/- mice, and HSC70 as the housekeeping loading control. Each lane represents a separate individual animal. (**B**) Quantification of FAK protein expression by densitometry. Expression levels of FAK in the livers of FAK-/- mice were <15% of that in WT mice. Sample size is n=3 per group, **p<0.01 by Student's t-test. Data represent individual data points and mean ± SEM.

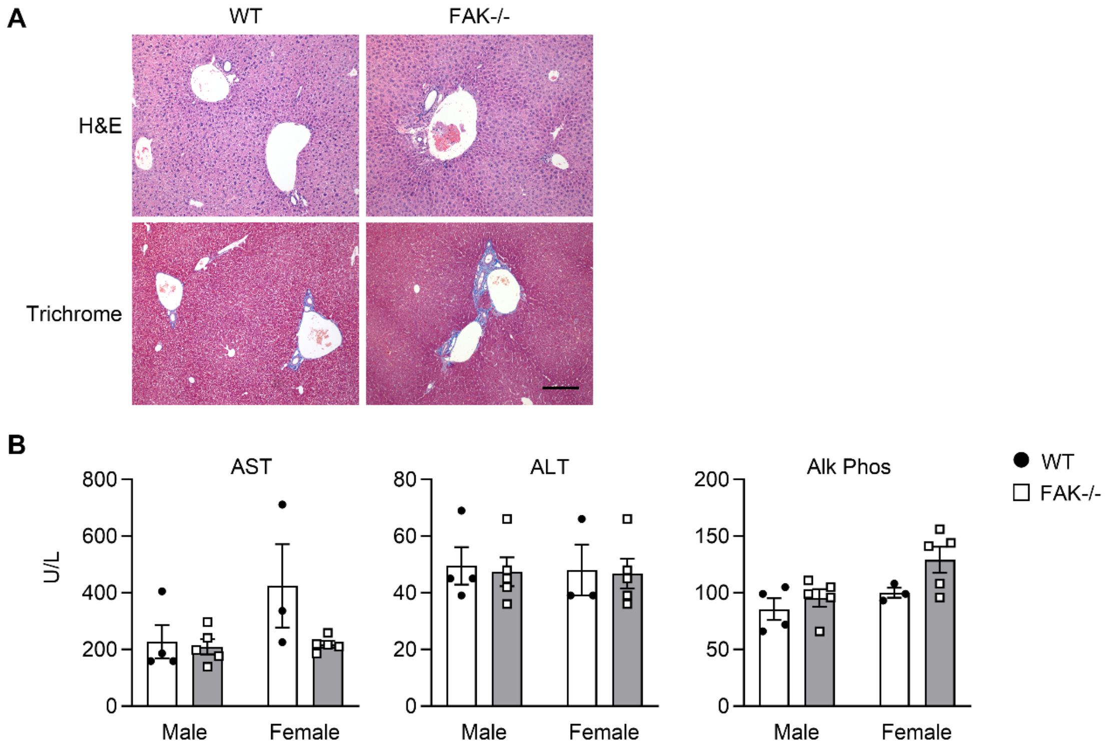

Supplemental Figure 2. Six-eight week-old FAK$^{fl/fl}$;Alb-cre^{+} mice (FAK-/-) mice show similar liver histology and serum liver functions tests compared to FAK$^{fl/fl}$;Alb-cre^{-} littermate controls (WT). (**A**) Representative liver H&E and trichrome histology of WT and FAK-/- mice. Scale bar=200μm. (**B**) Serum liver function tests: aspartate transaminase (AST), alanine transaminase (ALT), and alkaline phosphatase (Alk Phos). Sample sizes are WT male n=4, WT female n=3, FAK-/- male n=5, and FAK-/- female n=5. Data represent individual data points and mean ± SEM.

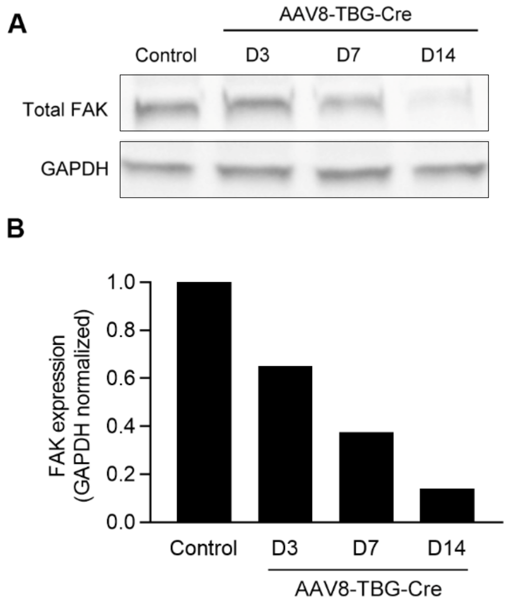

Supplemental Figure 3. FAK$^{fl/fl}$ mice treated with AAV8-TBG-Cre show efficient deletion of FAK in the liver after 2 weeks. (**A**) Western blot shows FAK protein expression in whole liver tissue lysate of FAK$^{fl/fl}$ mice (control) and FAK$^{fl/fl}$ mice treated with AAV8-TBG-Cre after 3, 7, or 14 days. GAPDH was used as the loading control. Each lane represents a separate individual animal. (**B**) Quantification of FAK protein expression by densitometry. Expression levels of FAK in the livers of AAV8-TBG-Cre-treated FAK$^{fl/fl}$ mice was <15% of that in control mice at 14 days.

Supplemental Table 1. qRT-PCR Primer Sequences.

Gene Name	Direction	Sequence 5'->3'	HBP[A] Primer ID
18s	Forward	GTGGAGCGATTTGTCTGGTT	NA[B]
	Reverse	CGCTGAGCCAGTCAGTGTAG	
Acta2	Forward	GGACGTACAACTGGTATTGTGC	NA[B]
	Reverse	TCGGCAGTAGTCACGAAGGA	
Col1a1	Forward	GCTCCTCTTAGGGGCCACT	NA[B]
	Reverse	CCACGTCTCACCATTGGGG	
Col1a2	Forward	GGTGAGCCTGGTCAAACGG	111120328c2
	Reverse	ACTGTGTCCTTTCACGCCTTT	
Col3a1	Forward	CTGTAACATGGAAACTGGGGAAA	20380522a1
	Reverse	CCATAGCTGAACTGAAAACCACC	
Col5a1	Forward	CTTCGCCGCTACTCCTGTTC	7656987a1
	Reverse	CCCTGAGGGCAAATTGTGAAAA	
Col6a1	Forward	CTGCTGCTACAAGCCTGCT	6753484a1
	Reverse	CCCCATAAGGTTTCAGCCTCA	
Col12a1	Forward	AGGCAGAAGTTGACCCACCT	111074528c1
	Reverse	CAGTGGTACTAGCTGCAAGGG	
Ihh	Forward	GACGAGGAGAACACGGGTG	31981670c2
	Reverse	GCGGCCCTCATAGTGTAAAGA	
Smo	Forward	GAGCGTAGCTTCCGGGACTA	26348965a1
	Reverse	CTGGGCCGATTCTTGATCTCA	
Gli2	Forward	CAACGCCTACTCTCCCAGAC	21411092a1
	Reverse	GAGCCTTGATGTACTGTACCAC	
Gli3	Forward	CACAGCTCTACGGCGACTG	6680021a1
	Reverse	CTGCATAGTGATTGCGTTTCTTC	
Ptch1	Forward	AAAGAACTGCGGCAAGTTTTTG	6679519a1
	Reverse	CTTCTCCTATCTTCTGACGGGT	

[A]Harvard Primer Bank. Identification numbers (ID) for primer sequences obtained from HPB are listed. (http://pga.mgh.harvard.edu/primerbank/citation.html; Wang X and Seed B. A PCR primer bank for quantitative gene expression analysis. Nucleic Acids Research. 2003;31(24): e154; pp.1-8.)

[B]Primers were designed using the online open-source Primer3 software. (Koressaar T, Lepamets M, Kaplinski L, Raime K, Andreson R and Remm M. Primer3_masker: integrating masking of template sequence with primer design software. Bioinformatics. 2018;34(11):1937-1938.)

Supplemental Methods

Western Blot

Whole liver lysates were prepared using Cell Extraction Buffer (Thermo Fisher Scientific, Waltham, MA) supplemented with phenylmethylsulfonyl fluoride and protease inhibitor cocktail (MilliporeSigma, St. Louis, MO). Total protein (25µg) was loaded onto 7.5% polyacrylamide gels for electrophoresis and wet transfer performed onto nitrocellulose membranes. Non-specific binding was blocked using 5% dry milk. Antibodies and dilutions used were total FAK (AHO0502 polyclonal; 1:200 Thermo Fisher Scientific), HSC70 (sc-7298; 1:5000; Santa Cruz Biotechnology, Dallas, TX) and GAPDH (14C10; 1:5000; Cell Signaling, Danvers, MA). Secondary antibodies conjugated with horse radish peroxidase (polyclonal; 1:5000; Jackson ImmunoResearch, West Grove, PA) were applied and chemiluminescent signal developed using the Pierce ECL2 Western Blotting Substrate (Thermo Fisher Scientific). Densitometry calculations were performed using ImageJ software.
